# Dataset of liver proteins of eu- and hypothyroid rats affected in abundance by any of three factors: in vivo exposure to hexabromocyclododecane (HBCD), thyroid status, gender differences

**DOI:** 10.1016/j.dib.2016.07.063

**Published:** 2016-08-05

**Authors:** I. Miller, J. Renaut, S. Cambier, A.J. Murk, A.C. Gutleb, T. Serchi

**Affiliations:** a Institute for Medical Biochemistry, Department for Biomedical Sciences, University of Veterinary Medicine Vienna, Veterinaerplatz 1, A-1210 Vienna, Austria; b Environmental Research and Innovation (ERIN) Department, Luxembourg Institute of Science and Technology (LIST), 5, Avenue des Hauts-Fourneaux, L-4362 Esch-sur-Alzette, Luxembourg; c Wageningen University, Marine Animal Ecology Group, De Elst 1, 6708 WD Wageningen, The Netherlands

**Keywords:** A1AT_RAT, Alpha-1-antiproteinase, ACOX1_RAT, Peroxisomal acyl-coenzyme A oxidase 1, AGT2_RAT, Alanine–glyoxylate aminotransferase 2_mitochondrial, AK1D1_RAT, 3-oxo-5-beta-steroid 4-dehydrogenase, AL1B1_RAT, Aldehyde dehydrogenase X_mitochondrial, BHMT1_RAT, Betaine–homocysteine S-methyltransferase 1, CAH3_RAT, Carbonic anhydrase 3, DOPD_RAT, D-dopachrome decarboxylase, FABPL_RAT, Fatty acid-binding protein_liver, FRIL1_RAT, Ferritin light chain 1, GRP78_RAT, 78 kDa glucose-regulated protein, GSTA1_RAT, Glutathione S-transferase alpha-1, GSTA2_RAT, Glutathione S-transferase alpha-2, GSTA3_RAT, Glutathione S-transferase alpha-3, GSTP1_RAT, Glutathione S-transferase P, HBA_RAT, Hemoglobin subunit alpha-1/2, HPT_RAT, Haptoglobin, K2C8_RAT, Keratin_type II cytoskeletal 8, KNT2_RAT, T-kininogen 2, M2GD_RAT, Dimethylglycine dehydrogenase_mitochondrial, PON1_RAT, Serum paraoxonase/arylesterase 1, PON3_RAT, Serum paraoxonase/lactonase 3, PRDX3_RAT, Thioredoxin-dependent peroxide reductase_mitochondrial, RD23B_RAT, UV excision repair protein RAD23 homolog B, RET1_RAT, Retinol-binding protein 1, SARDH_RAT, Sarcosine dehydrogenase_mitochondrial, SPA3N_RAT, Serine protease inhibitor A3N, UB2D2_RAT, Ubiquitin-conjugating enzyme E2 D2, HBCD, Proteomics, Rat, Liver, Hypothyroidism, Gender-specific effects

## Abstract

Male Wistar rats with different thyroid status (eu-, hypothyroid) were exposed to 0, 3 or 30 mg/kg body weight of the flame retardant HBCD for 7 days and obtained data compared with a previous study in females, “Hexabromocyclododecane (HBCD) induced changes in the liver proteome of eu- and hypothyroid female rats” (Miller et al., 2016) [1]. Specifically, proteomic investigation of liver protein patterns obtained by 2D-DIGE was performed and differences between animals groups recorded, based on the factors exposure, thyroid status and gender. All proteins with significantly changed abundance in any of these comparisons were identified by mass spectrometry. General, hormone and proteomic data of both the present and the previous studies are discussed in Miller et al. (2016) [1] and in "Gender specific differences in the liver proteome of rats exposed to hexabromocyclododecane (HBCD)" Miller et al. (2016) [2].

**Specifications Table**

**Table:** 

Subject area	*Biology*
More specific subject area	*Environmental Toxicology*
Type of data	*Tables, figure (PCA), image (annotated gel image)*
How data was acquired	*2D Fluorescence Difference Gel Electrophoresis (2D-DIGE) and mass spectrometry*
Data format	*Analyzed, filtered*
Experimental factors	*Liver lysates of eu- and hypothyroid male and female rats exposed to different concentrations of HBCD*
Experimental features	*Comparative proteomic analysis of rat liver lysates using 2D-DIGE. Proteins present in differentially abundant protein spots (regarding HBCD exposure, amount, thyroid status, and gender) were identified using MALDI TOF/TOF analysis.*
Data source location	*Origin of samples: Wageningen University, Wageningen, The Netherlands*
	*Data collection: Luxembourg Institute of Science and Technology, Esch-sur-Alzette, Luxembourg*
Data accessibility	*Data is with this article*

**Value of the data**

• Identification of baseline differences in the liver proteome between genders as the basis for future toxicological/pharmaceutical/mechanistic studies.
• Identification of liver proteins from male rats altered due to HBCD exposure or in hypothyroid status.
• Comparison of male and female rats exposed in a similar exposure experiment, but showing different responses in the liver proteome, will allow further targeting of proteins of interest.

## 1 Data

**Fig. 1:**
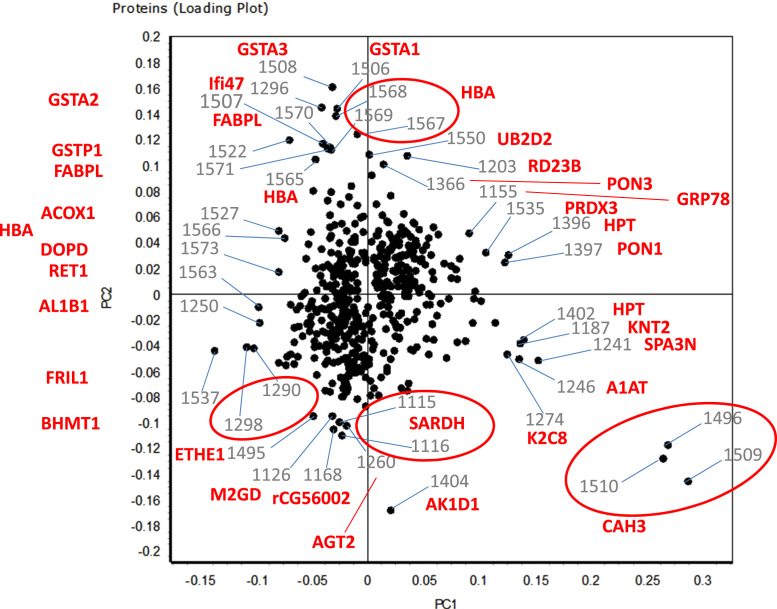
PCA Loading plot of the 496 differentially regulated spots. Spot numbers (corresponding to labeling in gel image in Suppl. Fig. 1 and regulation data in Suppl. Table 2) and UniProt accession numbers (Suppl. Table 1) used for labeling.

Patterns of 48 DIGE-gels with separations of liver protein lysates deriving from eu- and hypothyroid male and female rats of different HBCD exposures were evaluated. Differentially regulated spots in any of the group comparisons were subjected to MS identification, considering fold-changes of at least 30% between groups (*P*<0.05 within group) as statistically relevant (master gel with labels of all 496 identified spots in Suppl. Fig. 1; peptide identifications in Suppl. Table 1). Data from different animal groups in both genders were compared, taking into account the different aspects HBCD exposure, thyroid status, and gender (abundance changes in Suppl. Table 2; hierarchical clustering of regulated data in Suppl. Fig. 2; PCA in Fig. 1, with additional identification of single proteins in the border area or close to the main cluster).

## 2 Experimental design, materials and methods

### 2.1 Animals, treatment and experimental protocol

The animal experiment was detailed in [1], [2] and approved under number 2006-051 by the Animal Welfare Committee of Wageningen University. In brief, male Wistar WU (HsdCpbWU) rats with normal or reduced thyroid function (hypothyroid) were orally exposed to 0, 3 or 30 mg/kg bw/d HBCD, respectively, for 7 consecutive days. Four liver samples per group were analyzed by proteomic methods. The experimental setup was identical to the one used in [1], but applying it on male rats.

### 2.2 Proteomic analysis

Rat liver lysates were subjected to two-dimensional fluorescence difference gel electrophoresis (2D-DIGE) as previously described, without modifications, to make it compatible to the previously performed study in females [1], [2], [3]. This comprised a classical 2D-DIGE experiment, by separating CyDye-labeled proteins from liver lysates in a non-linear 3–10 pH-range and subsequently in large-format SDS-PAGE gels (260×200×1 mm). Gel images (acquired on a Typhoon 9400) were evaluated with the DeCyder 7.0 software package (both GE Healthcare, Diegem, Belgium), including all gels of the previous study on females in matching and statistics [1], [3]. Univariate and multivariate analyses were applied to highlight differentially regulated spots (fold change at least 1.3) with a *P*-value in the respective univariate ANOVA or two way ANOVA <0.05.

Spots differentially regulated in any of the group comparisons (including samples/gels from males and females) were automatically picked, trypsin digested and proteins identified by MALDI-TOF/TOF 5800 (Sciex) as previously described [1], [2], [3]. This also included spots from female rat livers previously not analyzed, as only now they were found regulated in the comparison with male rats׳ data. Protein identification was based on searches of mass fingerprints (PMF) and MS/MS spectra against the SwissProt database with “*Rattus norvegicus*” as taxonomy, using the ProteinPilot software (Sciex, Nieuwerkerk aan den Ijssel, The Netherlands) and the searching algorithm MASCOT (Matrix Science, www.matrixscience.com, London, UK). For each spot one protein mass fingerprint and up to 8 MS/MS spectra were generated. Parameters for the search were set as in the previous study [1], [3]: up to two missed cleavages allowed, 100 ppm tolerance in PMF, 0.75 Da mass tolerance for precursor ion mass, carbamidomethyl cysteine as fixed modification, oxidation of methionine and oxidation of tryptophan (single oxidation, double oxidation and kynurenine) as variable modifications. Identifications were considered to be significant when the combined MOWSE score had *P*<0.05.

For statistics the Extended Data Analysis (EDA) module as well as univariate analysis (ANOVA and *t*-test) and multivariate analysis (two way ANOVA), all part of the Decyder 7.0 software package, were applied.

## Appendix A Supplementary material

Supplementary material

Supplementary material Suppl. Table 1: protein identifications by MALDI-TOF/TOF analysis. For spot locations on the gel see gel image in Suppl. Fig. 1. Suppl. Table 2: All spots found affected in the complete/combined dataset (male and female rats). Ratios between different animal groups (*n*=4). Abbreviations: f, females; m, males; ET, euthyroid rats; HT, hypothyroid rats; 0, 3, 30 refer to amounts of HBCD (mg/kg bw/d) administered in the 7-day exposure period.
