# Supplementary material for: Dataset of liver proteins of eu- and hypothyroid rats affected in abundance by any of three factors: in vivo exposure to hexabromocyclododecane (HBCD), thyroid status, gender differences
Source: Data Brief. 2016 Aug 5;8:1344–7. doi: 10.1016/j.dib.2016.07.063 (PMC4992036; doi:10.1016/j.dib.2016.07.063)
Supplement: Supplementary file 1 — Supplementary material [file mmc1.doc]

Conflict of interest:

Authors do not declare any conflict of interest.

Ingrid Miller (on behalf of all authors)
